# Fault Detection and Exclusion for Tightly Coupled GNSS/INS System Considering Fault in State Prediction

**DOI:** 10.3390/s20030590

**Published:** 2020-01-21

**Authors:** Shizhuang Wang, Xingqun Zhan, Yawei Zhai, Baoyu Liu

**Affiliations:** 1 School of Aeronautics and Astronautics, Shanghai Jiao Tong University, Shanghai 200240, China; sz.wang@sjtu.edu.cn (S.W.); yawei.zhai@sjtu.edu.cn (Y.Z.); 2 Schulich School of Engineering, University of Calgary, Calgary, AB T2N 1N4, Canada; baoyu.liu@ucalgary.ca

**Keywords:** GNSS, INS, kalman filter, fault detection and exclusion, integrity monitoring

## Abstract

To ensure navigation integrity for safety-critical applications, this paper proposes an efficient Fault Detection and Exclusion (FDE) scheme for tightly coupled navigation system of Global Navigation Satellite Systems (GNSS) and Inertial Navigation System (INS). Special emphasis is placed on the potential faults in the Kalman Filter state prediction step (defined as “filter fault”), which could be caused by the undetected faults occurring previously or the Inertial Measurement Unit (IMU) failures. The integration model is derived first to capture the features and impacts of GNSS faults and filter fault. To accommodate various fault conditions, two independent detectors, which are respectively designated for GNSS fault and filter fault, are rigorously established based on hypothesis-test methods. Following a detection event, the newly-designed exclusion function enables (a) identifying and removing the faulty measurements and (b) eliminating the effect of filter fault through filter recovery. Moreover, we also attempt to avoid wrong exclusion events by analyzing the underlying causes and optimizing the decision strategy for GNSS fault exclusion accordingly. The FDE scheme is validated through multiple simulations, where high efficiency and effectiveness have been achieved in various fault scenarios.

## 1. Introduction

Tightly coupled navigation system of Global Navigation Satellite Systems (GNSS)/Inertial Navigation System (INS) is widely acknowledged as a suitable navigation solution for civil and military aircraft, aerial photogrammetry, Unmanned Aerial Vehicle (UAV), and Mobile Mapping Systems (MMS) [1]. It provides better performance than either GNSS or INS stand-alone system because it combines their advantages to give a continuous and complete navigation solution with high long- and short-term accuracy [2]. Additionally, tight integration achieves the balance between efficiency and performance in comparison with loose integration and deep integration [3].

Tight integration is usually implemented with an Extended Kalman Filter (EKF) which is a recursive estimator to generate optimal current-time state estimates in nominal cases [4]. However, the EKF-based integration system may produce large errors due to various faults in the system. The faults that affect current-time state estimates may exist in the measurements of GNSS and/or Inertial Measurement Unit (IMU), and may occur now and/or in the past.

In Safety-Critical Applications (SCAs), Fault Detection and Exclusion (FDE) is a critical aspect to prevent users from potential risk caused by rare sensor faults in navigation system. For example, the position failure of an aircraft or train may threaten the safety of the on-board passengers. And when the UAV or Urban Air Mobility (UAM) operates overhead the crowd for missions such as photogrammetry and cargo transportation [5], a navigation sensor fault may lead to catastrophic consequences for the ground pedestrians and constructions. In response, Fault Detection (FD) function provides the ability to alarm when faults occur in order to ensure integrity, and Fault Exclusion (FE) function enables excluding the faulty measurements to improve continuity [6].

The paper aims at developing an efficient and effective FDE scheme against sensor faults for tightly coupled GNSS/INS system. Inspired by literature [7] where sensor fault in state prediction is regarded as a threat to the navigation system, we revisit this problem with special emphasis on the potential faults in Kalman Filter state prediction step, besides the faults in current-time GNSS observations. As mentioned in [7], the fault (i.e., estimate bias) in state prediction, called filter fault (FF) in this paper, may be caused by (a) the faults in IMU measurements and (b) the undetected faults occurring prior to current time. Particularly, GNSS faults and FF exert negative impacts on the filter in significantly different ways: the former affects state update, while the latter influences state prediction. Accordingly, this difference should be highlighted in the design of FDE scheme.

FDE for GNSS-standalone navigation system in aviation applications has been exclusively studied in literature. For example, FDE is embedded in Receiver Autonomous Integrity Monitoring (RAIM) and Advanced RAIM (ARAIM) [8,9], where the navigation states are estimated by Least-Squares (LS) method. However, these techniques cannot be directly applied to the integrated system due to the significant difference in LS and EKF: the latter is a sequential approach while the former is a snapshot one.

Some FDE algorithms have been developed for tightly coupled GNSS/INS system over the past years. Autonomous Integrity Monitoring by Extrapolation [10], Innovation-Based (IB) method [11], Residual-Based (RB) method [11], Solution Separation (SS) method [12,13], Quality Control (QC) [14], Generalized Likelihood Ratio (GLR) [15], Extended RAIM (ERAIM) [16], and Rate Detector (RD) method [17] are the most representative hypothesis-test FDE algorithms. Recently, some novel methods have been proposed, including Support Vector Machine (SVM) approach [18], neural network approach [19], robust estimator approaches [20,21,22] and nonlinear filter approaches [23,24]. However, these novel methods aim to improve the performance of FD in terms of time-to-detect, but make it very difficult to evaluate the integrity risk (i.e., the probability of hazardously misleading information). In practice, integrity risk evaluation is important in SCAs because the navigation system is designed to satisfy expected levels of integrity and is required to provide the users with timely alerts when the system cannot meet the integrity requirements [25]. Accordingly, traditional hypothesis-test approaches are more suitable than these novel methods in SCAs because they are convenient and straightforward for error-bounding, probability calculation and integrity risk derivation.

The large majority of existing FDE schemes focused on the faults in the current epoch or in the sliding window. However, the undetected faults occurring previously also impact current-time innovations and state estimates because they introduce the estimate bias in state prediction in EKF. Ramp fault in pseudoranges, which represents a typical failure mode in GNSS, is one of the major sources of these undetected faults. To cope with the undetected faults, several sequential FD algorithms exploiting both current-time and past-time residuals were developed [4,26], and the sequential innovation-based detectors were also studied [27]. Although the undetected faults were taken into consideration in integrity risk evaluation in these methods, there were some obvious shortcomings that need to be addressed. First, the design of the FE algorithm was absent. Second, it has been proved that the innovation-based or residual-based fault detector would encounter performance degradation due to the estimate bias in state prediction caused by undetected faults [18]. Third, the sequential methods may be computationally expensive because they used a great number of innovations/residuals.

Moreover, the published algorithms shown above mainly aimed at detecting the faults in GNSS, without regard to IMU failures. Lee et al. proposed an integrity monitoring scheme for multi-sensor navigation system against IMU faults [7,28], but these studies were conducted from the perspective of protection level derivation rather than fault detection and exclusion. According to the mechanism of EKF, faults in IMU can also lead to the estimate bias in state prediction [28]. Despite the fact that Hardware Physics Redundancy (HPR) can significantly improve the reliability of IMU, it is necessary to pay due attention to the faults in IMU measurements. A significant number of serious accidents have happened due to IMU failures, such as the crashes of the Qantas F72 and Croatia Boeing 737-200 [29]. In addition, given that low-cost IMU sensors may be carried on UAVs for future UAM applications, such as passenger/cargo air transportation, the output of IMU must be monitored against the possible faults.

In this paper, we rigorously analyze the influence of FF on FDE and state estimates, and further propose an FDE algorithm to cope with both GNSS faults and FF. With FF being considered, special attentions should be attached to the mutual interference between GNSS faults and FF due to its adverse effects on fault detection performance. Accordingly, an FD scheme with parallel detectors, which are respective designated for GNSS fault and FF, is developed to separate their impacts. Then a two-step FE scheme including GNSS fault exclusion and filter recovery is presented. GNSS fault exclusion intends to remove the faulty measurements in GNSS and filter recovery aims to eliminate the estimate bias in state prediction. Regarding to GNSS fault exclusion, an optimized decision strategy is designed to improve the success rate of exclusion, and the mechanism of fault separation, a measure of the fault-exclusion capability, is investigated to reveal the underlying causes of wrong exclusion.

This paper is organized as follows. In Section 2, the mathematical model of GNSS/INS tight integration is described in detail considering various error sources and failure modes. With these analyses, we propose a novel fault detection scheme with two parallel detectors in Section 3. Then a two-step fault exclusion algorithm is presented in Section 4. In Section 5, simulations are carried out to validate the performance of the proposed algorithm in various fault scenarios. Finally, Section 6 concludes the paper and presents some perspectives.

## 2. Tightly Coupled GNSS/INS Integration

### 2.1. Tightly Coupled GNSS/INS Integration Model

**Figure 1:**
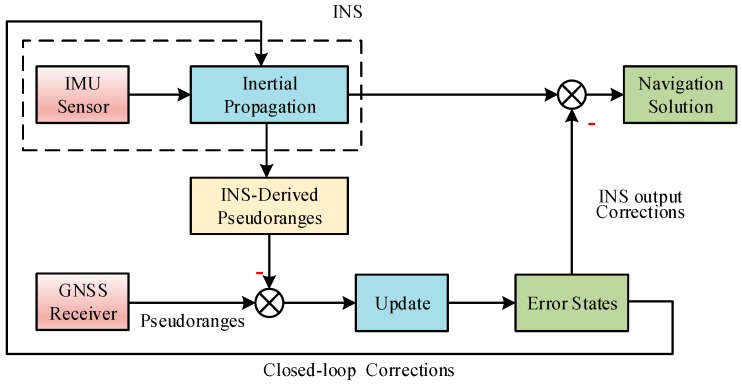
Closed-loop error-state Global Navigation Satellite Systems (GNSS)/Inertial Navigation System (INS) tight integration architecture.

In this paper, tightly coupled GNSS/INS integration is implemented by adopting a closed-loop error-state EKF. The architecture is illustrated in Figure 1. Here, the errors estimated by EKF are fed back every iteration to correct the system itself, maintaining a linear approximation in the system model [1].

The main target of GNSS/INS tight integration is to correct the INS solution and to compensate the inertial sensor errors, with GNSS measurements as external aiding. Let $N_{const}$ be the number of GNSS constellations used in the integration. Then, the $\left(15+N_{const}+1\right)$-parameter error state in the filter is shown as follows [3]:

(1) $$x={\left[\;{\left(\delta \phi \right)}^{T},\;{\left(\delta v\right)}^{T},{\left(\delta p\right)}^{T},\nabla_{a}^{T},\nabla_{g}^{T},\delta \rho_{u},\delta {\dot{\rho }}_{u}\right]}^{T}$$

where δϕ, δv, and δp denote the INS error states of attitude, velocity, and position, respectively; $\nabla_{a}$ and $\nabla_{g}$ are the accelerometer and gyro bias vectors; $\delta \rho_{u}$ and $\delta {\dot{\rho }}_{u}$ respectively represent the GNSS receiver clock state vectors, i.e., $N_{const}$ pseudorange biases caused by clock offset regarding each constellation and 1 pseudorange rate bias for receiver clock drift. Specifically, the IMU bias drift in each direction is modelled as a first-order Gauss-Markov process in the filter.

The discrete-time process model and measurement model are shown in Equations (2) and (3), respectively.

(2) $$x_{k}=\Phi_{k|k-1}x_{k-1}+\omega_{k}$$

(3) $$z_{k}=H_{k}x_{k}+\nu_{k}$$

where $\Phi_{k|k-1}$ is the state transition matrix from epoch (k−1) to epoch k;$\;H_{k}$ denotes the measurement matrix; $z_{k}$ is the measurement vector; and $\omega_{k}$ and $\nu_{k}$ are the process noise vector and measurement noise vector, respectively. Both $\omega_{k}$ and $\nu_{k}$ are modeled as Gaussian White Noise (GWN), with process noise covariance matrix $Q_{k}$ and measurement noise covariance matrix $R_{k}$, respectively.

In EKF, prediction and update are two essential steps to obtain an optimal state vector estimate. As shown in (4) and (5), the prediction step is used to predict the state vector *x* and the associated covariance matrix *P* from the last to the current epoch, using the assumed process model.

(4) $${\hat{x}}_{k}^{-}=\Phi_{k|k-1}{\hat{x}}_{k-1}^{+}$$

(5) $$P_{k}^{-}=\;\Phi_{k|k-1}P_{k-1}^{+}\Phi_{k|k-1}^{T}+Q_{k}$$

where the superscripts “−” and “+” represent a predicted (a prior) estimate and an updated (a posterior) estimate, respectively.

As shown in Equations (6) and (7), the update step is used to update the state vector and the associated covariance matrix according to the measurement model.

(6) $${\hat{x}}_{k}^{+}=\;{\hat{x}}_{k}^{-}+K_{k}r_{k}$$

(7) $$P_{k}^{+}=\left(I-K_{k}H_{k}\right)P_{k}^{-}$$

where $K_{k}$ is the Kalman gain matrix and $r_{k}$ is the innovation vector. The Kalman gain matrix $K_{k}$ is determined as:

(8) $$K_{k}=\;P_{k}^{-}H_{k}^{T}{\left(H_{k}P_{k}^{-}H_{k}^{T}+R_{k}\right)}^{-1}$$

The innovation vector is defined as the difference between the actual measurement vector and the predicted one as shown in Equation (9).

(9) $$r_{k}=\;z_{k}-H_{k}{\hat{x}}_{k}^{-}$$

When EKF operates in a closed-loop mode, the predicted state vector ${\hat{x}}_{k}^{-}$ is zero because of the feedback process [1]. As a result:

(10) $$r_{k}=z_{k}=\;\rho_{G}-\rho_{I}$$

where $\rho_{G}$ is the GNSS pseudorange vector and $\rho_{I}$ is the predicted pseudorange vector from INS-derived navigation solution (before update) [3]. If the filter works optimally (i.e., fault-free), the innovation sequence $r_{k}$ is a zero-mean GWN, whose covariance matrix $V_{k}$ is given as [3]:

(11) $$V_{k}=H_{k}P_{k}^{-}H_{k}^{T}+R_{k}$$

**Figure 2:**
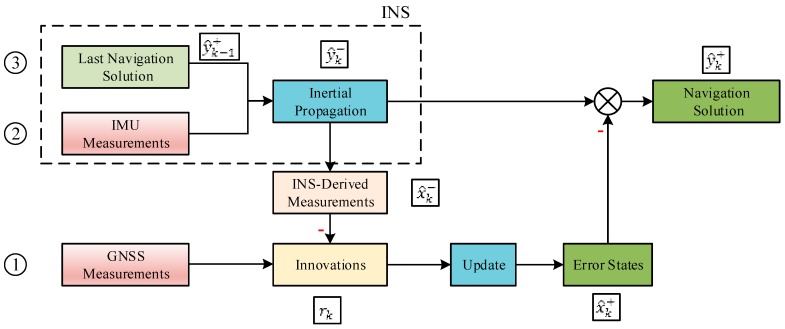
Prediction process and update process in the integrated system. (Note, x—error states, and y —absolute states).

In addition to the error states, we should also pay attention to the absolute states (i.e., attitude, velocity, position, IMU sensor biases, and receiver clock states) for the following reasons. First, the navigation system intends to provide the navigation solution rather than the error states to the users. Second, the predicted measurements $\rho_{I}$ and the innovations $r_{k}$ depend on the predicted absolute navigation information. To illustrate the relationship between the error states and the absolute states (labeled as y), the system-propagation (prediction) process and the measurement-update (update) process in GNSS/INS tight integration are shown in Figure 2.

### 2.2. Error Analysis of Tight Integration Model

Navigation errors are unavoidable due to various errors (noises) in the prediction and update steps of the filter. According to Figure 2, these errors can be divided into three parts: GNSS pseudorange errors, IMU measurement errors, and last navigation solution errors. Table 1 presents a brief introduction to the errors that affect the current-time state estimate, including their causes and models (a detailed illustration is given in [30]).

According to Figure 2, both the noises in last navigation solution and those in IMU measurements lead to the errors in INS-derived navigation solution and therefore impact the INS-derived measurements. Based on these analyses, the simplified mathematical models of GNSS pseudorange vector $\rho_{G}$ and the predicted pseudorange vector $\rho_{I}$ are given as follows:

(12) $$\rho_{G}=\;\rho_{R}+\varepsilon_{G}$$

(13) $$\rho_{I}=\rho_{R}+\varepsilon_{I}=\rho_{R}+G\cdot \varepsilon_{y}$$

where $\rho_{R}$ is the noise-free (true) pseudorange vector; $\varepsilon_{G}$ is the pseudorange noise vector; $\varepsilon_{I}$ represents the INS-derived pseudorange noise vector; $\varepsilon_{y}$ is the noise vector of INS-derived navigation solution; and G is the geometry matrix of the observations [1]. Substituting Equations (12) and (13) into (10), we can get:

(14) $$r_{k}=\;\rho_{G}-\rho_{I}=\varepsilon_{G}-G\cdot \varepsilon_{y}$$

### 2.3. Fault Analysis of Tight Integration Model

The navigation system can occasionally encounter significantly large output errors due to the potential faults in the integrated system. Similar to the errors, the faults that impact current-time state estimates can be divided into three parts as shown in Table 2.

According to Figure 2 and Table 2, current-time GNSS faults impact the state update step, while faults in IMU measurements or in the last navigation solution lead to the bias in INS-derived navigation solution (i.e., state prediction step). The bias is called “filter fault (FF)” in this paper as stated in the Introduction. All the recursive effects of the undetected faults occurring previously are represented by FF. It is justified because it is filter fault and current-time GNSS faults that directly impact the current-time innovations and state estimates. As a result, taking the faults into consideration, $\rho_{G}$ and $\rho_{I}$ can be modeled as:

(15) $$\rho_{G}=\;\rho_{R}+\varepsilon_{G}+b_{G}$$

(16) $$\rho_{I}=\;\rho_{R}+G\cdot \left(\varepsilon_{y}+b_{y}\right)$$

where $b_{G}$ denotes the GNSS fault vector and $b_{y}$ is the vector of the bias in INS-derived navigation solution (i.e., FF). Both $b_{G}$ and $b_{y}$ represents the effects of various faults on current-time innovations and state estimates. Then the innovation vector is re-written as:

(17) $$r_{k}=\varepsilon_{G}+b_{G}-G\cdot \varepsilon_{y}-G\cdot b_{y}$$

Equation (17) illustrates the effects of noises and faults on the innovations and lays the foundation of the design of FDE schemes. Their effects on the navigation solution are given in Appendix A.

## 3. FG-AIME: A Novel Fault Detection Scheme with Two Detectors

### 3.1. Fault Detection Based on AIME

AIME is a typical FD scheme for tightly coupled GNSS/INS system, with better performance in detecting ramp faults than the snapshot methods [10]. AIME exploits the sequential innovations in the siding window of length *l* to build the chi-squared test statistic.

First, batch processing is used to determine the averaged innovation vector $r_{avg}$ as follows:

(18) $$r_{avg}={\left(V_{avg}^{-1}\right)}^{-1}\overset{k}{\underset{i=k-l+1}{\sum }}V_{i}^{-1}r_{i}$$

where $V_{avg}^{-1}$ denotes the inverse of the averaged innovation covariance matrix, determined by:

(19) $$V_{avg}^{-1}=\overset{k}{\underset{i=k-l+1}{\sum }}V_{i}^{-1}$$

Second, the test statistic is established as follows:

(20) $$s_{avg}=r_{avg}^{T}\left(V_{avg}^{-1}\right)r_{avg}$$

The statistic $s_{avg}$ follows a central chi-squared distribution whose degrees of freedom (DOF) is equal to the number of visible satellites $N_{sat}$ in fault-free cases, and it follows a non-central chi-squared distribution in fault scenarios [10].

Third, the detection threshold $T_{A}$ is determined based on the probability of false alarm $P_{FA}$ which is obtained from the navigation performance requirements. For example, this value is 8 × 10^−6^ per approach in LPV-200 [9]. The threshold is determined by:

(21) $$1-P_{FA}=F(T_{A}|N_{sat})$$

where $F(*|N_{sat})$ denotes the cumulative distribution function (CDF) of the central chi-squared distribution with $N_{sat}$ DOF. If$\;s_{avg}>T_{A}$, a fault alert is raised.

To further interpret AIME, we take an eigenvalue-decomposition perspective. Since the covariance matrix $V_{k}$ is symmetric and positive definite, $V_{k}$ can be expressed as:

(22) $$V_{k}=L\cdot {\hat{V}}_{k}\cdot L^{-1}$$

where L denotes an orthogonal matrix of eigenvectors, and ${\hat{V}}_{k}$ is the diagonal eigenvalue matrix whose eigenvalues are all positive. Then,

(23) $${\hat{r}}_{k}=L^{T}\cdot r_{k}$$

where ${\hat{r}}_{k}$ denotes the transformed innovation vector whose covariance matrix is ${\hat{V}}_{k}$. Hence, the elements in ${\hat{r}}_{k}$ are uncorrelated with each other. The test statistic$\;s_{k}$ is given as:

(24) $$s_{k}={\left({\hat{r}}_{k}\right)}^{T}\cdot \left({\hat{V}}_{k}^{-1}\right)\cdot {\hat{r}}_{k}=r_{k}^{T}\cdot \left(V_{k}^{-1}\right)\cdot r_{k}$$

Equations (22)–(24) prove that $s_{avg}$ follows $N_{sat}$-DOF chi-squared distribution. A detailed proof is provided in [10].

### 3.2. Enhanced AIME Scheme Based on Fault Grouping

AIME is designed to detect GNSS faults and it will experience performance degradation when FF occurs due to the interference between GNSS faults and FF as shown in Equation (17). To separate the effects of GNSS faults and FF, an enhanced AIME method, FG-AIME, is proposed based on fault grouping, which comprises parallel GNSS fault detector and FF detector.

First, the test statistic in GNSS fault detector is constructed as follows. According to Equation (17), an innovation-based vector unaffected by FF is obtained by:

(25) $${\tilde{r}}_{G}=\left(I-G\cdot S\right)\cdot r_{k}=\left(I-G\cdot S\right)\cdot \left(\varepsilon_{G}+b_{G}\right)$$

where I denotes the $N_{sat}$-by-$N_{sat}$ identity matrix and S is the least squares coefficient matrix defined as:

(26) $$S={\left(G^{T}G\right)}^{-1}\cdot G^{T}$$

Then we calculate the corresponding covariance matrix of ${\tilde{r}}_{G}$ as:

(27) $${\tilde{V}}_{G}=\left(I-G\cdot S\right)\cdot V_{k}\cdot {\left(I-G\cdot S\right)}^{T}$$

Using Equation (11), it is easy to prove that:

(28) $${\tilde{V}}_{G}=\left(I-G\cdot S\right)\cdot R_{k}\cdot {\left(I-G\cdot S\right)}^{T}$$

It is worth noting that ${\tilde{V}}_{G}$ is singular with rank of$\;\left(N_{sat}-3-N_{const}\right)$ [33]. Consequently, before building the test statistic, eigenvalue decomposition of ${\tilde{V}}_{G}$ must be performed:

(29) $${\tilde{V}}_{G}=L_{G}\cdot {\hat{V}}_{G}\cdot L_{G}^{-1}$$

where $L_{G}$ denotes the orthogonal matrix of eigenvectors and ${\hat{V}}_{G}$ is the diagonal eigenvalue matrix. The eigenvalues comprise $\left(N_{sat}-3-N_{const}\right)$ non-zero elements and $\left(3+N_{const}\right)$ zero elements (including 3 position elements and $N_{const}$ receiver clock element). We define $L_{G}^{e}$ and ${\hat{V}}_{G}^{e}$ as the parts, which are corresponding to non-zero eigenvalues, of $L_{G}$ and ${\hat{V}}_{G}$, respectively. Then we transform ${\tilde{r}}_{G}$ into$\;{\tilde{r}}_{G}^{e}$ by:

(30) $${\tilde{r}}_{G}^{e}={\left(L_{G}^{e}\right)}^{T}\cdot {\tilde{r}}_{G}$$

The test statistic $s_{avg}^{G}$ in GNSS fault detector is constructed with ${\tilde{r}}_{G}^{e}$ and ${\hat{V}}_{G}^{e}$ in the same way as AIME approach (i.e., Equations (18)–(20)). This statistic follows a central and non-central chi-squared distribution with $\left(N_{sat}-3-N_{const}\right)$ DOF in fault-free cases and faulty cases, respectively.

Second, the test statistic in FF detector is built as follows. The innovation-based vector ${\tilde{r}}_{F}$ for FF detection is obtained as:

(31) $${\tilde{r}}_{F}=S\cdot r_{k}=S\cdot \left(\varepsilon_{G}+b_{G}\right)-\varepsilon_{y}-b_{y}$$

**Figure 3:**
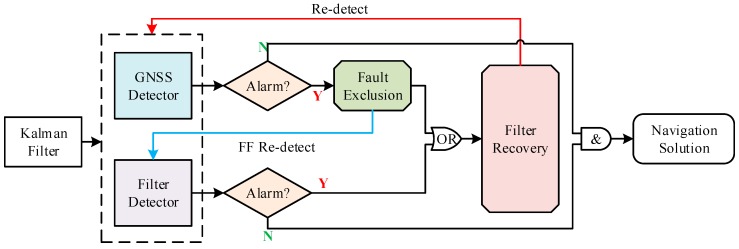
The architecture of the proposed Fault Detection and Exclusion (FDE) scheme.

Unfortunately, as shown in Equation (31), ${\tilde{r}}_{F}$ is affected by GNSS faults because which satellites are faulty is unknown before FE. The test statistic based on ${\tilde{r}}_{F}$ reflects, therefore, the effects of both GNSS faults and FF. After excluding the faulty satellites in FE, the FF detector should be repeated using the new innovation vector free of the effects of GNSS faults (see Figure 3 in Section 4.1).

Then we calculate the corresponding covariance matrix of ${\tilde{r}}_{F}$ as:

(32) $${\tilde{V}}_{F}=S\cdot V_{k}\cdot S^{T}$$

where ${\tilde{V}}_{F}$ is also singular, whose rank is $\left(3+N_{const}\right)$. The derivation of test statistic $s_{avg}^{F}$ in FF detector is the same as that in AIME, and it is omitted for the sake of brevity.

In FG-AIME, $P_{FA}$ should be properly allocated to the two fault detectors. A simple principle for the allocation is provided: $P_{FA}^{F}$ should be high if the prior probability of FF is larger than that of GNSS faults, e.g., using low-cost IMU in the system; otherwise $P_{FA}^{G}$ should be high to enhance the FD capability for GNSS faults. Specially, only GNSS faults will be detected when$\;P_{FA}^{G}=P_{FA}$. Therefore, the adjustable allocation of $P_{FA}$ indeed enhances the adaptability of the proposed FD scheme to various scenarios. In fact, the optimal allocation should be determined considering both the FD capability and the integrity risk, but this is beyond the scope of this paper.

## 4. Fault Exclusion with Two Steps: GNSS Fault Exclusion and Filter Recovery

### 4.1. Complete Fault Detection and Exclusion Scheme

A complete architecture of the proposed FDE scheme is shown in Figure 3. For the purpose of ensuring navigation continuity, the proposed FE scheme performs two functions: excluding the faulty satellites, and recovering the filter after GNSS fault exclusion or FF detector’s alarm. Filter recovery represents the process of correcting the estimate bias in state prediction (i.e., $b_{y}$), which will be illustrated in detail in Section 4.3. Additionally, as stated in Section 3.2., the FF detector should be repeated after GNSS fault exclusion. Finally, after the entire FE process, FD should be repeated to make sure that the system has no fault alarm before outputting the navigation solution.

### 4.2. GNSS Fault Exclusion: Statistics and Decision Strategy

#### 4.2.1. Alternative Hypotheses and Statistics for GNSS Fault Exclusion

**Figure 4:**
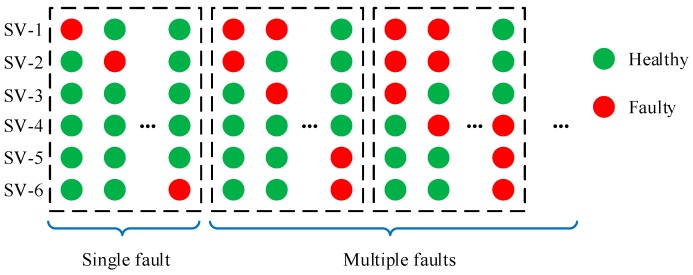
An example of various subsets ($N_{sat}=6$).

GNSS fault exclusion is attempted when GNSS fault detector alarms. There are a set of alternative hypotheses for exclusion. Each of them is linked to a subset which labels the satellites as faulty/healthy according to the associated hypothesis. Figure 4 provides an example of various subsets.

To avoid calculating the statistics for all the subsets, we pre-set the probability $P_{ignored}$ accounting for unconsidered fault modes to filter out the subsets with low probabilities. Then the maximum number $N_{max}$ of simultaneous faults that need to be considered is determined based on the prior fault probability of each subset. The method to determine $N_{max}$ and the monitored subsets is provided in ARAIM baseline algorithm description [9]. 

For each subset determined above, the statistic is constructed based on the corresponding hypothesis. For subset j, a new innovation-based vector is given as:

(33) $${\tilde{r}}_{G}^{\left(j\right)}=\left(I-G\cdot S^{\left(j\right)}\right)\cdot r_{k}$$

where $S^{\left(j\right)}$ denotes the least squares coefficient matrix corresponding to subset j, determined by:

(34) $$S^{\left(j\right)}={\left({\left(G^{\left(j\right)}\right)}^{T}G^{\left(j\right)}\right)}^{-1}\cdot {\left(G^{\left(j\right)}\right)}^{T}$$

where $G^{\left(j\right)}$ denotes the geometry matrix for subset j. It is obtained by substituting the rows in G, which is corresponding to the satellites labeled as faulty in subset j, with vectors whose entries are all zero.

Substituting (17) into (33), we get:

(35) $${\tilde{r}}_{G}^{\left(j\right)}=\left(I-G\cdot S^{\left(j\right)}\right)\cdot \left(\varepsilon_{G}+b_{G}\right)$$

According to Equation (35), ${\tilde{r}}_{G}^{\left(j\right)}$ is only affected by the noises and faults in GNSS measurements. Let $A^{\left(j\right)}=\left(I-G\cdot S^{\left(j\right)}\right)$ for brevity. Then we define $r_{H}^{\left(j\right)}$ as the elements of ${\tilde{r}}_{G}^{\left(j\right)}$ corresponding to the satellites labeled as healthy in subset j, which is determined by:

(36) $$r_{H}^{\left(j\right)}=A_{H}^{\left(j\right)}\cdot \left(\varepsilon_{G}+b_{G}\right)$$

where $A_{H}^{\left(j\right)}$ takes the corresponding rows of$\;A^{\left(j\right)}$. We then compute the covariance matrix $V_{H}^{\left(j\right)}$ of $r_{H}^{\left(j\right)}$ as:

(37) $$V_{H}^{\left(j\right)}=A_{H}^{\left(j\right)}\cdot V_{k}\cdot {\left(A_{H}^{\left(j\right)}\right)}^{T}=A_{H}^{\left(j\right)}\cdot R_{k}\cdot {\left(A_{H}^{\left(j\right)}\right)}^{T}$$

where $V_{H}^{\left(j\right)}$ is singular with a rank of$\;\left(N_{sat}-3-N_{const}-N_{F}^{\left(j\right)}\right)$ and $N_{F}^{\left(j\right)}$ is the number of satellites assumed faulty in subset j.

The test statistic ${\bar{s}}_{FE}^{\left(j\right)}$ of subset j for fault exclusion is constructed based on $r_{H}^{\left(j\right)}$ and $V_{H}^{\left(j\right)}$ in the same way as the derivation of$\;s_{avg}^{G}$. If all the faulty satellites are labeled as faulty in subset j, each element of $r_{H}^{\left(j\right)}$ should follow a zero-mean Gaussian distribution and therefore ${\bar{s}}_{FE}^{\left(j\right)}$ should follow a central chi-squared distribution. Otherwise, ${\bar{s}}_{FE}^{\left(j\right)}$ should follow a non-central chi-squared distribution.

#### 4.2.2. Decision Strategy for GNSS Fault Exclusion

To allow the navigation service to continue without loss of continuity in fault scenarios, FE is required to accurately exclude the faulty measurements. Consequently, when designing the GNSS fault exclusion scheme, we should take special measures to avoid wrong exclusions, including over exclusion and incomplete exclusion, as shown in Table 3. The proposed decision strategy for GNSS fault exclusion with the use of the statistics determined above is illustrated below and a flowchart of the algorithm implementation is provided for summarization.

If GNSS fault detector alarms, a set of satellites of size $N_{e}$ should be excluded. A basic strategy to determine the best candidate set of satellites for exclusion is provided in ARAIM [9], which is shown as follows. For each possible size $N_{e}$ of the set, we determine the best candidate for exclusion by finding the subset with the smallest chi-squared statistic [9]:

(38) $$j_{N_{e}}=\underset{j}{\mathrm{argmin}}\{{\bar{s}}_{FE}^{\left(j\right)}|N_{F}^{\left(j\right)}=N_{e}\}$$

For the candidate subset $\;j_{N_{e}}$, a chi-squared test should be performed for consistency check [9], and the threshold $T_{G}^{\left(new\right)}$ is computed as:

(39) $$1-P_{FA}^{G}=F\left(T_{G}^{\left(new\right)}|N_{sat}-3-N_{const}-N_{e}\right)$$

The subset is deemed to pass the test when ${\bar{s}}_{FE}^{\left(j_{N_{e}}\right)}\leq T_{G}^{\left(new\right)}$. The search for the effective candidate for exclusion starts from $N_{e}=1$ and stops when the best candidate of $N_{e}$ passes the test above.

**Figure 5:**
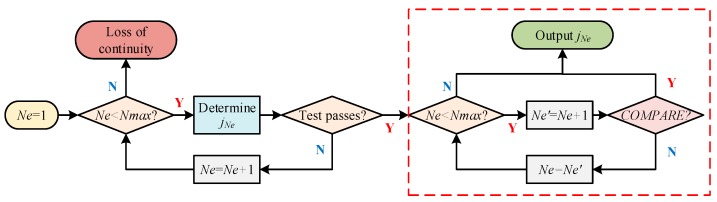
Flowchart of the determination of best candidate for GNSS fault exclusion.

In addition to the previous process, extra measures should be taken to reduce the probability of wrong exclusion. We provide an efficient approach based on the comparison between the candidate subsets $j_{N_{e}}$ and $j_{N_{e}+1}$ for this purpose. Figure 5 presents the complete process of determining the best candidate for GNSS fault exclusion. In Figure 5, the procedures in the red-dotted box are used to cope with wrong exclusion, where the COMPARE module aims to determine whether there is over exclusion or incomplete exclusion.

The output of the COMPARE module is Y only when the following two statements are both true:

1. All the satellites labeled as faulty in subset $j_{N_{e}}$ are labeled as faulty in subset $j_{N_{e}{}^{\prime }}$;
2. The difference of the statistics Δs between subset $j_{N_{e}}$ and subset $j_{N_{e}{}^{\prime }}$ is smaller than the corresponding threshold $T_{\Delta s}$. The determination of Δs and $T_{\Delta s}$ is given in Appendix B.

In this module, statement 1 is used to determine whether healthy satellites are excluded in $j_{N_{e}}$ and statement 2 is to avoid over exclusion. Detailed illustration will be given in Section 5.3.

#### 4.2.3. Analysis of Fault Separation Problem

Fault separation represents the separability [16] between two fault modes. Fault separation intends to quantitatively analyze the possibility of the event that satellite 2 is flagged as faulty when fault actually occurs in satellite 1. Fault separation also attempts to reveal the underlying causes of wrong exclusion, which is significant for FE performance improvement in future researches.

Assuming hypothesis $j_{0}$ is right, then the statistic ${\bar{s}}_{FE}^{\left(j_{0}\right)}$ follows a central chi-squared distribution and ${\bar{s}}_{FE}^{\left(j\right)}$ follows a non-central chi-squared distribution when$\;N_{F}^{\left(j\right)}\leq N_{F}^{\left(j_{0}\right)}\left(j\neq j_{0}\right)$. Based on this, we present an indicator to reveal the separability between hypothesis $j_{0}$ and hypothesis j as follows.

An innovation-based vector for hypothesis j is given by:

(40) $${\tilde{r}}^{\left(j\right)}={\tilde{L}}^{\left(j\right)}\cdot r_{H}^{\left(j\right)}={\tilde{L}}^{\left(j\right)}\cdot A_{H}^{\left(j\right)}\cdot \left(\varepsilon_{G}+b_{G}\right)$$

where ${\tilde{L}}^{\left(j\right)}$, a $\left(N_{sat}-3-N_{const}-N_{F}^{\left(j\right)}\right)$-by-$\left(N_{sat}-N_{F}^{\left(j\right)}\right)$ transformation matrix, satisfies:

(41) $${\tilde{L}}^{\left(j\right)}\cdot V_{H}^{\left(j\right)}\cdot {\left({\tilde{L}}^{\left(j\right)}\right)}^{T}={\tilde{V}}^{\left(j\right)}$$

where ${\tilde{V}}^{\left(j\right)}$ is a diagonal matrix whose diagonal elements are non-zero eigenvalues of$\;{\tilde{V}}_{H}$. Based on Equation (24), the non-central parameter$\;\delta^{\left(j\right)}$ is determined by:

(42) $$\delta^{\left(j\right)}={\left({\tilde{L}}^{\left(j\right)}\cdot A_{H}^{\left(j\right)}\cdot b_{G}\right)}^{T}\cdot {\left({\tilde{V}}^{\left(j\right)}\right)}^{-1}\cdot \left({\tilde{L}}^{\left(j\right)}\cdot A_{H}^{\left(j\right)}\cdot b_{G}\right)$$

For single-fault scenarios, a correlation coefficient $C_{j_{0}}^{j}$ for fault separation independent of fault magnitude is calculated as:

(43) $$C_{j_{0}}^{j}={\left({\tilde{L}}^{\left(j\right)}\cdot A_{H}^{\left(j\right)}\cdot q^{\left(j_{0}\right)}\right)}^{T}\cdot {\left({\tilde{V}}^{\left(j\right)}\right)}^{-1}\cdot \left({\tilde{L}}^{\left(j\right)}\cdot A_{H}^{\left(j\right)}\cdot q^{\left(j_{0}\right)}\right)$$

where $q^{\left(j_{0}\right)}$ represents the normalized fault vector for hypothesis$\;j_{0}$, i.e., $q^{\left(j_{0}\right)}={\left[0,0,\ldots ,1,0,\ldots 0\right]}^{T}$. Note that the coefficients for multi-faults scenarios can also be obtained based on Equation (43) but they are dependent on the fault amplitude ratios among different satellites.

According to the decision strategy for GNSS fault exclusion given above, i.e., Equation (38), hypothesis j may be misjudged as right if $C_{j_{0}}^{j}$ or $\delta^{\left(j\right)}$ is small because the effect of $\delta^{\left(j\right)}$ on the difference between ${\bar{s}}_{FE}^{\left(j_{0}\right)}$ and ${\bar{s}}_{FE}^{\left(j\right)}$ may be subtle compared to that of random noises. So, $C_{j_{0}}^{j}$ is an indicator to reveal the separability between hypotheses, and the bigger it is, the lower the probability of the misjudgment will be.

### 4.3. Filter Recovery After GNSS Fault Exclusion

After excluding the faulty satellites, filter recovery attempts to correct the estimate bias $b_{y}$ (i.e., FF). In this section, two filter recovery schemes are proposed, including bias compensation method and re-filter method. The schemes presented here are preliminary and should be viewed as examples of filter recovery for the integrated system.

First, as shown in Figure 3, the FF detector should be repeated after GNSS fault exclusion as follows:

(44) $$r_{F}^{\prime }=S^{\left(j_{e}\right)}\cdot r_{k}=S^{\left(j_{e}\right)}\cdot \left(\varepsilon_{G}+b_{G}\right)-\varepsilon_{y}-b_{y}$$

(45) $$V_{F}^{\prime }=S^{\left(j_{e}\right)}\cdot V_{k}\cdot {\left(S^{j_{e}}\right)}^{T}$$

where $j_{e}$ corresponds to the best candidate for GNSS fault exclusion determined in Section 4.2. Given that the hypothesis $j_{e}$ is very likely to be right, $r_{F}^{\prime }$ will be unaffected by GNSS faults, i.e., $S^{\left(j_{e}\right)}\cdot b_{G}=0$. Consequently, FF detector will only reflect FF. The new test statistic ${\bar{s}}_{F}^{\prime }$ in FF detector is determined based on $r_{F}^{\prime }$ and $V_{F}^{\prime }$ in the same way as $s_{avg}^{F}$.

Second, the filter recovery schemes are given as follows. The bias compensation method is to compensate the INS-derived navigation solution ${\hat{y}}_{k}^{-}$ using the estimate ${\hat{b}}_{y}$ of the estimate bias $b_{y}$. Based on (44), we have:

(46) $${\hat{b}}_{y}=-r_{F}^{\prime }$$

The re-filter method is to store the historical measurements in a preceding horizon of m epochs and re-run the filter from epoch (k−m+1) to current epoch k with faulty satellites excluded. Appendix C presents the method to properly determine the length of the preceding horizon. However, the re-filter method will be unavailable when faults occur in the unique IMU sensor. To cope with these cases, new fault exclusion strategies will be investigated for the integrated system with redundant IMU sensors in future work.

## 5. Simulation and Discussion

### 5.1. Simulation Description

A simulation platform of EKF-based tightly coupled GNSS/INS system is built to demonstrate the proposed FDE scheme. The simulation parameters of IMU (aviation-grade) and GNSS measurements are shown in Table 4 [1].

Various faults are added to GNSS pseudoranges and IMU raw measurements as shown in Table 5. Ramp fault with slope of 0.1 m/s is a typical failure type in GNSS pseudoranges [31]. The step faults added to the accelerometer are also justified for the following reasons. First, using closed-loop integration architecture, slowly growing errors (i.e., ramp faults) exert little influence on both the navigation solution and the innovations because IMU sensor errors are estimated and fed back to INS for corrections every epoch. Second, step faults of about 0.2 m/s^2^ in IMU may be caused by a sudden change of the constant biases, which is possible, especially for low-cost sensors [34].

**Figure 6:**
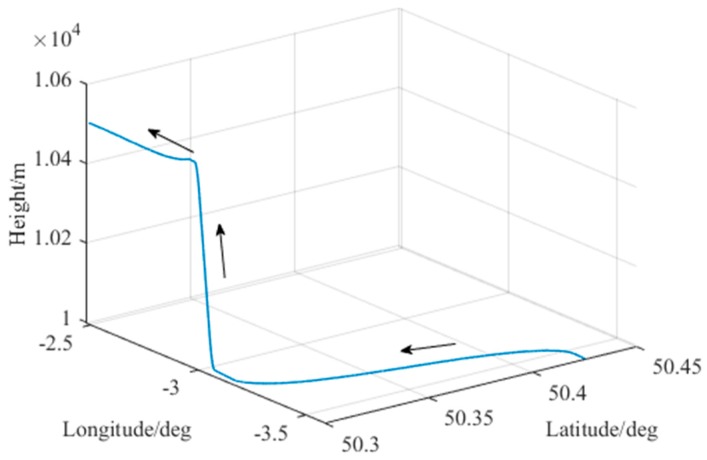
Simulated trajectory of the aircraft.

As shown in Figure 6, the trajectory in this simulation is of a 418-s aircraft motion generated by Spirent SimGen, in a speed of 200 m/s with two 45° turns in opposite directions and a 500 m climb [1].

### 5.2. Fault Detection Based on AIME and FG-AIME

This section demonstrates the proposed FD scheme in various fault scenarios. In the following simulations, $P_{FA}$ is set to 8e-6 (see [9]) and the length of sliding window l (see Section 3.1) is set to 10 s.

**Figure 7:**
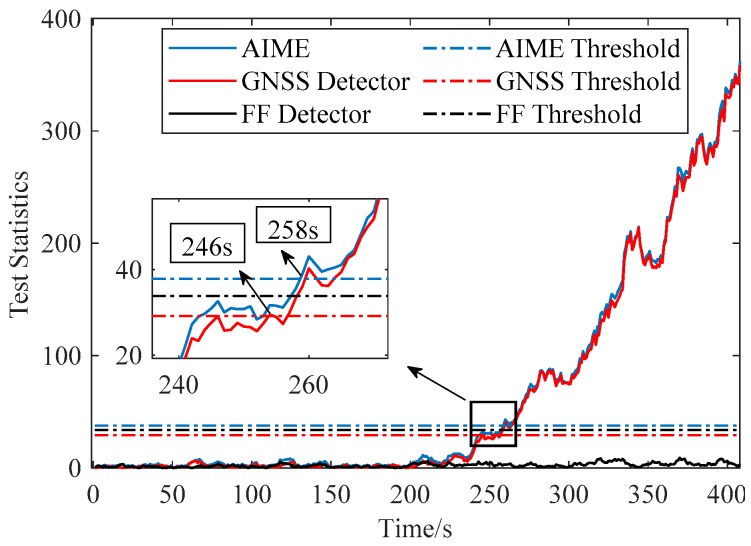
Fault detection for GNSS ramp fault occurring in SV-3 ($P_{FA}^{G}=0.9P_{FA}$).

**Figure 8:**
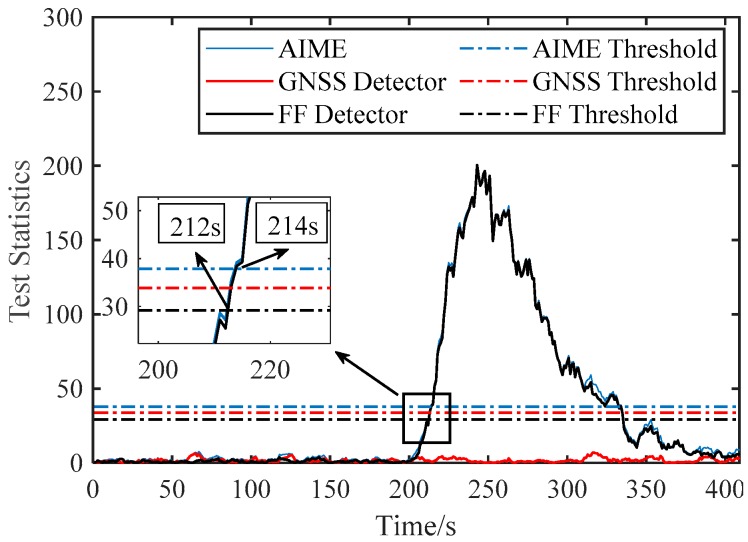
Fault detection for step faults occurring in accelerometer ($P_{FA}^{F}=0.9P_{FA}$).

Figure 7 and Figure 8 compare the performance of AIME and FG-AIME in GNSS fault scenario (No. 1 in Table 5) and IMU fault scenario (No. 3 in Table 5), respectively. Regarding the performance on timely detection, results show that FG-AIME is superior to AIME with the detection delay reduced by 12 s and 2 s, respectively. In Figure 8, the decrease of the test statistic in FF detector is caused by the gradual closed-loop correction process for the step faults in IMU. Additionally, Figure 8 proves that IMU faults should also be monitored because they can issue a fault alarm and may lead to large navigation errors. Note that, 90% of $P_{FA}$ is allocated to GNSS fault detector in Figure 7 (scenario No. 1) to enhance the detection capability of GNSS faults, while the major part of $P_{FA}$ is assigned to FF detector in Figure 8 because we assume that an unreliable IMU is used and IMU faults are considered in the simulation (scenario No. 3).

**Figure 9:**
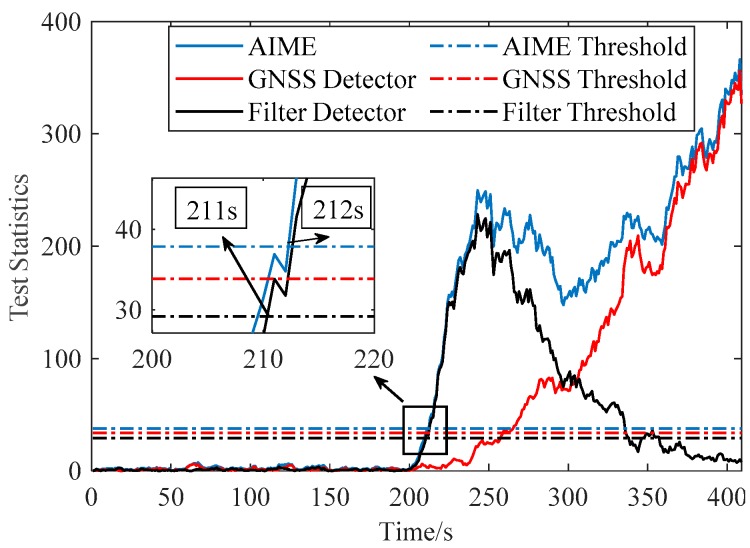
Fault detection for faults in both SV-1 and accelerometer ($P_{FA}^{F}=0.9P_{FA}$).

Figure 9 evaluates the performance of FG-AIME when GNSS faults and IMU faults occur simultaneously (scenario No. 4). Regarding the detection delay, the performance of AIME and FG-AIME is quite similar. However, we cannot conclude that FG-AIME is of little significance in this case. As mentioned earlier, the motivation of FG-AIME is to separate the effects of GNSS faults and FF. This is beneficial for the two detectors in FG-AIME to reflect the faults more accurately. Furthermore, the information of fault sources provided by FG-AIME is essential for FE to determine whether to exclude GNSS satellites and whether to perform filter recovery. Moreover, from the perspective of integrity monitoring, this information is also important for integrity risk evaluation because the effects of GNSS faults and FF on the position errors are quite different [28], which is derived in detail in Appendix A.

### 5.3. GNSS Fault Exclusion, Fault Separation, and Filter Recovery

**Figure 10:**
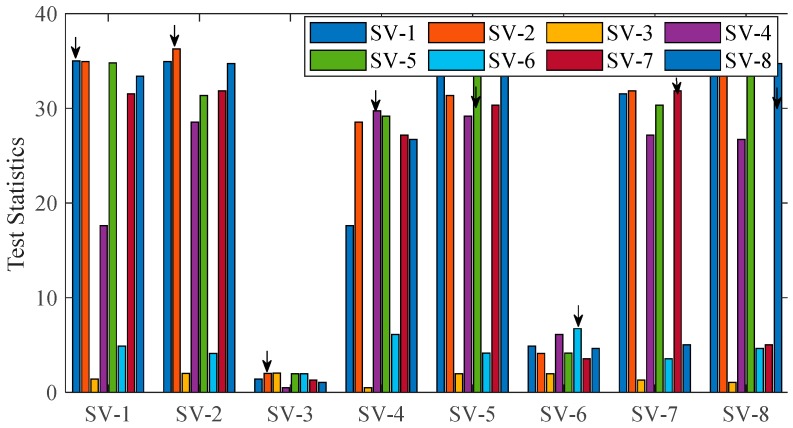
Statistics for GNSS fault exclusion when fault occurs in SV-3.

**Figure 11:**
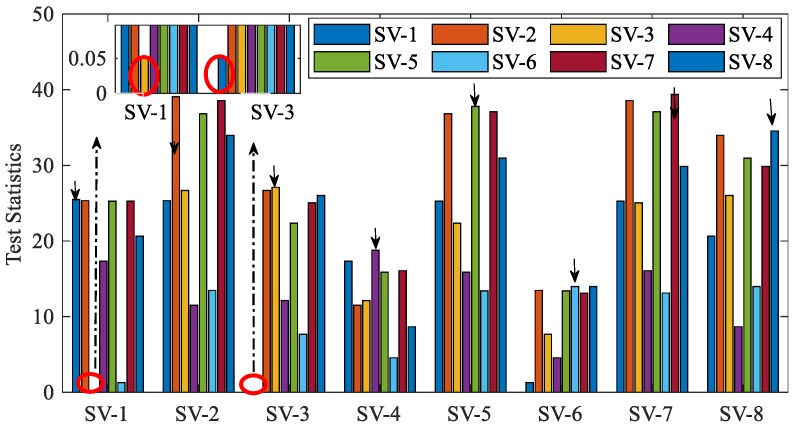
Statistics for GNSS fault exclusion when faults occur in SV-1 and SV-3.

This section demonstrates the FE scheme in various fault scenarios. Notes for Figure 10 and Figure 11 are given first. One X-axis tag (SV-I) and one legend tag (SV-J) form a hypothesis (SV-I, SV-J). Both SV-I and SV-J are assumed faulty in dual-fault hypothesis (SV-I, SV-J, I ≠ J) or hypothesis (SV-J, SV-I, I ≠ J). Only SV-I is assumed faulty in single-fault hypothesis (SV-I, SV-I) which is marked by black arrow in the figures.

Figure 10 shows the instantaneous statistics for GNSS fault exclusion in a single-fault case (i.e., SV-3 is faulty) when the detector starts to alarm (see Figure 7). The following process is an illustration of Figure 5, i.e., the flowchart of GNSS fault exclusion. Based on Equation (38), the best candidate $j_{1}$ is hypothesis (SV-3, SV-3) among single-fault hypotheses (i.e., $N_{e}=1$). This candidate can pass the consistency-check test because the threshold is 26.6 for $N_{e}=1$ based on Equation (39). Then, extra steps are taken to avoid wrong exclusion: the best candidate $j_{2}$ among dual-fault hypotheses is hypothesis (SV-3, SV-4) or (SV-4, SV-3) and the difference $\Delta s=\left({\bar{s}}_{FE}^{\left(j_{1}\right)}-{\bar{s}}_{FE}^{\left(j_{2}\right)}\right)$ is significantly small, which shows that SV-4 is healthy and excluding SV-3 is sufficient. Therefore, SV-3 will be excluded, which is obviously a right exclusion.

Figure 11 displays the instantaneous statistics in a dual-fault case (i.e., SV-1 and SV-3 are faulty, see scenario No. 2 in Table 5). Among single-fault hypotheses, the best candidate $j_{1}$ is hypothesis (SV-6, SV-6). This candidate can pass the consistency-check test because the threshold is 26.6 as mentioned earlier. If there are no extra steps, SV-6 will be excluded, which is apparently a wrong exclusion. The extra steps are given as follows. The best candidate $j_{2}$ is hypothesis (SV-1, SV-3) or (SV-3, SV-1). According to the COMPARE module in Figure 5, candidate $j_{2}$ is the best candidate for exclusion because Statement 1 is false, i.e., the satellite (i.e., SV-6) assumed faulty in candidate $j_{1}$ is assumed healthy in candidate$\;j_{2}$. Therefore, SV-1 and SV-3 will be excluded, which is a right exclusion. Accordingly, Figure 11 proves the effectiveness of the proposed GNSS FE scheme in avoiding wrong exclusion.

**Figure 12:**
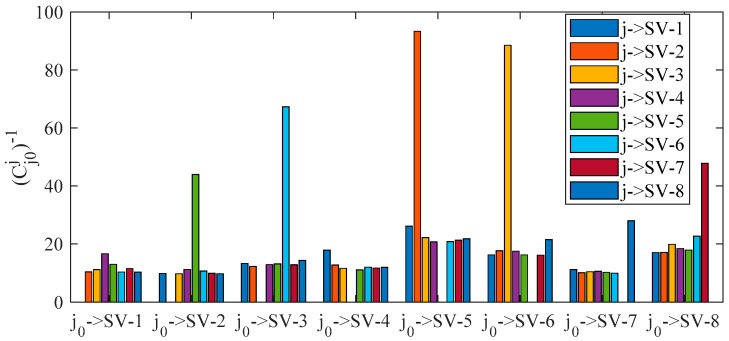
Reciprocal of correlation coefficient in single-fault cases for fault separation evaluation.

To illustrate the concept of fault separation, Figure 12 presents the reciprocal of correlation coefficient in single-fault cases. In this figure, we predefine that hypothesis $j_{0}$ is true and hypothesis j is false (*j* ≠ *j*_0_). The larger the coefficient ${\left(C_{j_{0}}^{j}\right)}^{-1}$ is, the higher the probability of misjudging hypothesis j as right will be. For example, it is hard to determine whether to exclude SV-3 or SV-6 when ramp fault occurs in SV-3 because ${\left(C_{j_{0}\to SV3}^{j\to SV6}\right)}^{-1}$ is large and then the statistic corresponding to hypothesis (SV-6, SV-6) will be small, which has been proved in Figure 10.

**Figure 13:**
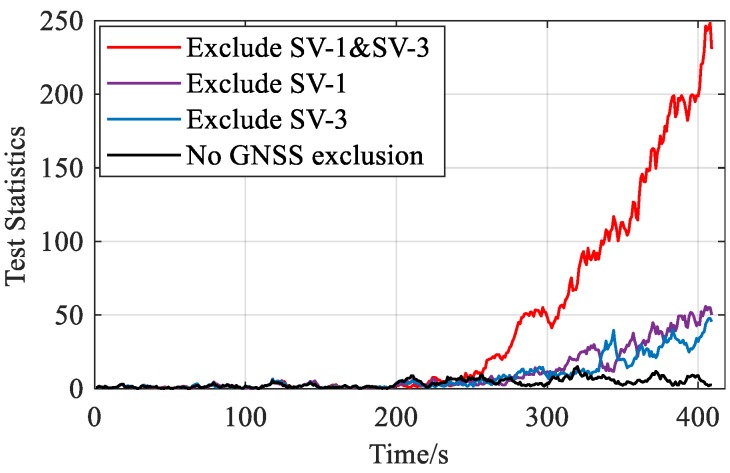
Chi-squared test statistics in filter fault (FF) detector before/after GNSS fault exclusion.

FF detection should be repeated after GNSS fault exclusion to eliminate the effects of GNSS faults. Figure 13 shows the chi-squared statistics in FF detector before and after GNSS fault exclusion when faults occur in SV-1 and SV-3. As shown in Equation (31), the innovation-based vector ${\tilde{r}}_{F}$ is affected by GNSS fault, and thus the test statistics cannot accurately reflect the magnitude of filter fault before GNSS fault exclusion. As shown in Equation (34), the effects of GNSS faults are eliminated by excluding the faulty satellites when constructing the new vector $r_{F}^{\prime }$. When there is a right exclusion, i.e., excluding both SV-1 and SV-3, FF can be accurately reflected by the statistic. This also proves that the faults occurring previously may lead to the noticeable estimate bias. In contrast, if there is a wrong exclusion or no exclusion, the test statistics will be affected by GNSS faults and cannot reflect the estimate bias accurately. Consequently, Figure 13 indeed indicates the importance of repeating FF detection step after GNSS FE.

**Figure 14:**
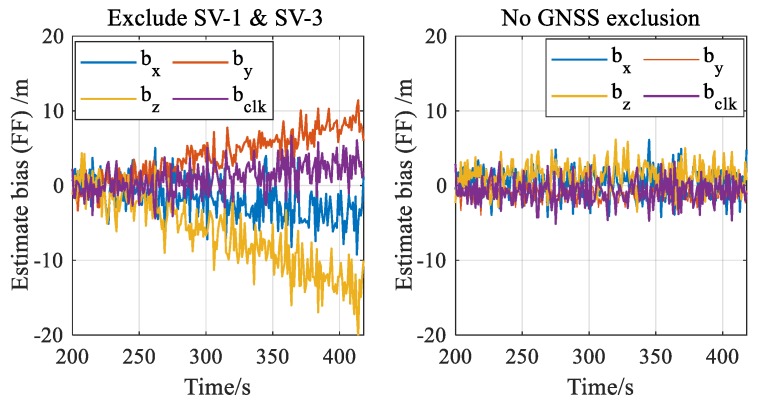
Estimation of the estimate bias (FF) when faults occur in SV-1 and SV-3 (left: estimation after excluding SV-1 and SV-3; right: no exclusion).

To further reveal the underlying causes of Figure 13, Figure 14 compares the estimate ${\hat{b}}_{y}$ of the estimate bias $b_{y}$, including 3 position components and 1 receiver clock component, before and after GNSS FE when faults occur in SV-1 and SV-3. The associated chi-squared statistics in Figure 13 are calculated based on these components and the covariance matrix given in Equations (32) and (35). Noticeable differences between the two subfigures are shown in Figure 14, where the right subfigure reflects the estimate bias (i.e., $b_{y}$) more accurately owing to the elimination of GNSS faults’ interference by excluding the faulty satellites (SV-1 and SV-3). Without GNSS exclusion, the estimate is heavily “polluted” by the effects of GNSS faults, and it cannot track the true status of filter fault consequently. As a result, it is necessary to estimate the magnitudes of filter fault after GNSS fault exclusion for both fault detection and filter recovery.

**Figure 15:**
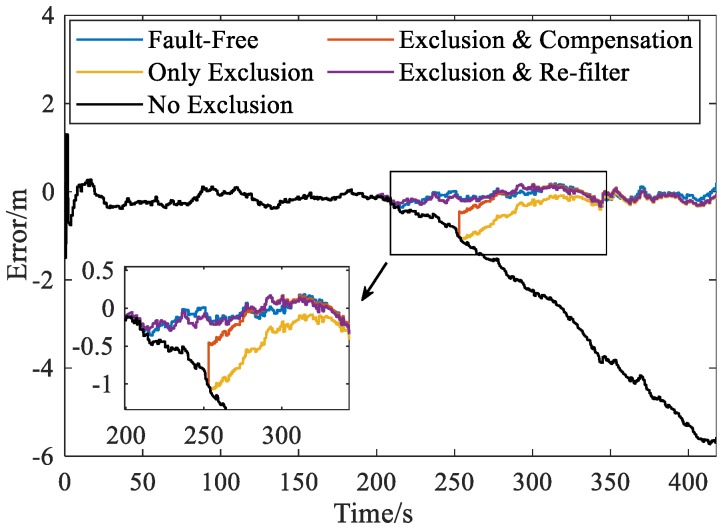
Position errors (north) with different filter recovery strategies.

Filter recovery is used to remove the estimate bias in the filter. Figure 15 provides the comparison of north position errors using different filter recovery strategies when fault occurs in SV-3. Results show that both the bias compensation method (“Exclusion and Compensation”) and the re-filter method (“Exclusion and Re-filter”) are conducive to reducing the navigation error after GNSS fault exclusion. Note that, when using the re-filter method, we simply re-run the integration process from 200 s with SV-3 excluded to show the performance of this method. Actually, as mentioned in Section 4.3, the start epoch of the re-filter process can be optimally determined by the method provided in Appendix C. Figure 15 also verifies the importance of FE by comparing the errors with and without exclusion: the position errors increase continuously when the faulty measurements are not excluded from the system (“No Exclusion” curve) while errors are greatly reduced when the proposed FDE scheme is employed in fault scenario.

## 6. Conclusions and Prospects

This paper presents a comprehensive Fault Detection and Exclusion (FDE) scheme for tightly coupled navigation system of Global Navigation Satellite Systems (GNSS)/Inertial Navigation System (INS), with special emphasis on the fault in state prediction (called filter fault). The scheme is beneficial to ensuring navigation safety of civil and military aircraft, Unmanned Aerial Vehicle (UAV), etc. Theoretical analyses quantitatively reveal the different effects of GNSS faults and filter fault on the filter, which motivates the design of an effective FDE scheme to handle these faults independently. Accordingly, the Fault Detection (FD) scheme, comprising two detectors, is designed to separate the effects of GNSS faults and filter fault. And the two-step Fault Exclusion (FE) scheme, with GNSS fault exclusion and filter recovery, is developed to exclude the faulty satellites and eliminate the effects of filter fault. Multiple simulations are conducted to validate the new scheme in various fault scenarios. The results show that: (a) this scheme excels in detecting single or multiple GNSS faults, filter fault, and simultaneous faults in both GNSS and Inertial Measurement Unit; (b) the FE scheme can accurately identify and exclude the faulty satellites with the optimized decision strategy, and the filter recovery scheme shows promising performance in eliminating the effects of filter fault; (c) the indicators for fault-exclusion capability analysis quantify the difficulty in identifying the faulty satellites, which actually reveals the underlying causes of wrong exclusion.

Possible improvements and future work include: (a) integrity risk derivation for the integrated system; (b) design of new FDE scheme with redundant IMUs; (c) FE capability enhancement based on the analysis of fault separation.

## Figures and Tables

**Table 1:** Sources, causes, and models of the errors in the integration system.

Sources	Causes	Models
GNSS pseudoranges	They are caused by Signal-In-Space errors, ionosphere and troposphere propagation delay, multipath and receiver noise, etc.	The measurement noise is modeled as zero-mean GWN with covariance matrix *R_k_*.
IMU measurements	For high-end IMU, only bias drift and random noises should be considered ^1^.	Bias drift is modeled as a first order Gauss-Markov process and is included in the error states; random noises are modeled as zero-mean GWN and their covariance is given in *Q_k_*.
Last navigation solution	The noises in last navigation solution are caused by the noises in previous prediction and update steps.	The noises are described by a zero-mean multi-dimensional Gaussian distribution, whose covariance matrix is *P_K_*_−1_.

^1^ For low-cost inertial sensors, the turn-on bias and scale factor should also be considered and they should be included in the error states.

**Table 2:** Sources, causes, and types of the faults in the integration system.

Sources	Causes	Types
GNSS pseudoranges (labeled as $b_{G}$)	They are caused by satellite clock jump, clock drift, incorrect ephemeris, etc.	Typical fault types include: ramp faults and step faults [31].
IMU measurements (included in $b_{y}$)	IMU faults, including bias instability and scale-factor non-linearity, gyro drift, etc., may occur due to various internal and external causes, e.g., mechanical failures, abnormal temperature, excessive humidity, severe vibration, etc. [32].	Typical faults in IMU are in the form of ramp faults, step faults, periodic faults, and constant output.
Last navigation solution (included in $b_{y}$)	The faults in last navigation solution are caused by the undetected faults occurring prior to current time, including the previous faults in IMU and GNSS.	The type of this fault is related to the types and duration of the previous faults in GNSS and IMU [28], and it can be stepped, ascending or descending.

**Table 3:** Possible results in GNSS fault exclusion process.

Results	Exclude All Faulty Satellites?	Exclude Any Healthy Satellites?
Right exclusion	YES	NO
Over exclusion	YES	YES
Incomplete exclusion	NO	YES/NO

**Table 4:** Simulation parameters.

Sensors	Parameter	Value
IMU	Accelerometer noise root PSD ^1^	$20\;\mu g/\sqrt{Hz}$
	Gyro noise root PSD	$0.002\;{}^{\circ}/\sqrt{hr}$
	Accelerometer biases (body frame)	[30;−45;26] μg
	Gyro biases (body frame)	[−0.0009; 0.0013; 0.0008] °/*hr*
	Output rate	100 Hz
GNSS	Pseudorange noise (1σ)	2.5 m
	Output rate	1 Hz
	Number of visible satellites	8

^1^ PSD: power spectral density.

**Table 5:** Fault scenarios in the simulations.

No.	Sources	Fault Information	Fault Duration
1	GNSS	Add 0.1 m/s ramp fault to SV-1 or SV-3 ^1^	200 s-end
2		Add 0.1 m/s ramp faults to SV-1 and SV-3	
3	IMU	Add 0.2 m/s^2^ step faults to each accelerometer axis	
4	GNSS&IMU	Add 0.1m/s ramp fault to SV-1 and 0.2 m/s^2^ step faults to each accelerometer axis	

^1^ SV: Space Vehicle = Satellite.

## Appendix A. Faults’ Effects on the Navigation Solution

Based on the fault analysis of the integration model in Section 2.3, the faults’ effects on the navigation solution are derived. According to Figure 2, the navigation solution is given as:

(A1) $${\hat{y}}_{k}^{+}={\hat{y}}_{k}^{-}+{\hat{x}}_{k}^{+}={\hat{y}}_{k}^{-}+K_{k}\cdot r_{k}$$

Substituting Equation (17) into (A1), we have:

(A2) $${\hat{y}}_{k}^{+}=y_{k}+\varepsilon_{y}+b_{y}+K_{k}\cdot \left(\varepsilon_{G}+b_{G}-H_{k}\cdot \varepsilon_{y}-H_{k}\cdot b_{y}\right)$$

where $y_{k}$ is the true navigation solution. Then, Equation (A2) can be re-written as:

(A3) $${\hat{y}}_{k}^{+}=y_{k}+\left(I-K_{k}\cdot H_{k}\right)\cdot \left(\varepsilon_{y}+b_{y}\right)+K_{k}\cdot \left(\varepsilon_{G}+b_{G}\right)$$

Equations (A2) and (A3) reveal the effects of GNSS faults and filter fault on the navigation solution, and they also prove the necessity of considering FF and separating the effects of these two faults in fault detection. Additionally, these equations are the foundations of integrity risk derivation.

## Appendix B. Determination of Δs and $T_{\Delta s}$ in COMPARE Module

The difference of the statistics between subset $j_{1}$ and subset $j_{2}$, i.e., $\Delta s=\left({\bar{s}}_{FE}^{\left(j_{1}\right)}-{\bar{s}}_{FE}^{\left(j_{2}\right)}\right)$, is used to determine whether the candidate $j_{1}$ is an incomplete exclusion. According to the rank one update formula [9], we have:

(A4) $$\Delta s={\bar{s}}_{FE}^{\left(j_{1}\right)}-{\bar{s}}_{FE}^{\left(j_{2}\right)}=\frac{1}{1-g_{i}^{T}\cdot {\left({\left(G^{\left(j_{1}\right)}\right)}^{T}G^{\left(j_{1}\right)}\right)}^{-1}\cdot g_{i}}\times {\left(r_{k}^{\left(j_{1}\right)}\left[i\right]\right)}^{2}$$

where i represents the index of the satellite which is assumed faulty in hypothesis $j_{2}$ but healthy in hypothesis$\;j_{1}$; $g_{i}$ is the $i^{th}$ row of$\;G^{\left(j_{1}\right)}$; and $r_{k}^{\left(j_{1}\right)}\left[i\right]$ is the $i^{th}$ entry of$\;r_{k}^{\left(j_{1}\right)}$, which forms a normal distribution. Based on Equation (A4), we get:

(A5) $$\Delta s=\Upsilon \cdot \bar{\Delta s}$$

where $\bar{\Delta s}$ follows 1-DOF central chi-squared distribution when satellite i is healthy and Υ is a coefficient derived from Equation (A4). Then the corresponding threshold $T_{\Delta s}$ can be obtained based on the CDF of 1-DOF central chi-squared distribution as shown in Equation (21).

## Appendix C. Determination of the Optimal Preceding Horizon Length for Re-Filter Method

First, we derive the expectation of the navigation errors caused by current and previous faults. Based on Equation (A3), the expectation $\mu_{k}$ is determined as:

(A6) $$\mu_{k}=\left(I-K_{k}\cdot H_{k}\right)\cdot b_{yk}+K_{k}\cdot b_{Gk}$$

Substituting Equation (2) into Equation (A6), we have:

(A7) $$b_{yk}=\Phi_{k|k-1}\mu_{k-1}+b_{Ik}$$

where $b_{Ik}$ is the IMU fault vector. Then,

(A8) $$\mu_{k}=\left(I-K_{k}\cdot H_{k}\right)\cdot \Phi_{k|k-1}\cdot \mu_{k-1}+\left(I-K_{k}\cdot H_{k}\right)\cdot b_{Ik}+K_{k}\cdot b_{Gk}$$

Let $A_{k}=\left(I-K_{k}\cdot H_{k}\right)$ and $B_{k}=\left(I-K_{k}\cdot H_{k}\right)\cdot \Phi_{k|k-1}$, we get:

(A9) $$\mu_{k}=\left(\overset{k-m+1}{\underset{i=k}{\prod }}B_{i}\right)\mu_{k-m}+\overset{1}{\underset{t=m}{\sum }}\left(\left(\overset{k-t+1}{\underset{i=k}{\prod }}B_{i}\right)\left(A_{k-t}\cdot b_{I\left(k-t\right)}+K_{k-t}\cdot b_{G\left(k-t\right)}\right)\right)+A_{k}\cdot b_{Ik}+K_{k}\cdot b_{Gk}$$

where m is the length of the preceding horizon that needs to be determined.

The first two terms on the right-hand side of Equation (A9) reflect the effects of the navigation solution bias in epoch (k−m) and the faults in the preceding horizon, respectively. It has been proved that $\left(\prod_{i=k}^{k-m+1}B_{i}\right)\cdot \mu_{k-m}$ tends to decrease as m becomes large [35]. If m=k, this term will be 0. Obviously, the determination of the optimal length m shows a tradeoff between the complexity and the residual errors, i.e., $\left(\prod_{i=k}^{k-m+1}B_{i}\right)\cdot \mu_{k-m}$. So, the optimal length should be determined considering both the computational payload and the acceptable residual errors.
